# Meditation-related activations are modulated by the practices needed to obtain it and by the expertise: an ALE meta-analysis study

**DOI:** 10.3389/fnhum.2012.00346

**Published:** 2013-01-04

**Authors:** Barbara Tomasino, Sara Fregona, Miran Skrap, Franco Fabbro

**Affiliations:** ^1^Dipartimento di Scienze Umane, Università di UdineUdine, Italy; ^2^Corso di Laurea in Psicologia, Università di TriesteTrieste, Italy; ^3^Unità Operativa di Neurochirurgia, A.O.S. Maria della MisericordiaUdine, Italy

**Keywords:** meditation, expertise, fMRI, ALE meta-analysis, attention

## Abstract

The brain network governing meditation has been studied using a variety of meditation practices and techniques practices eliciting different cognitive processes (e.g., silence, attention to own body, sense of joy, mantras, etc.). It is very possible that different practices of meditation are subserved by largely, if not entirely, disparate brain networks. This assumption was tested by conducting an activation likelihood estimation (ALE) meta-analysis of meditation neuroimaging studies, which assessed 150 activation foci from 24 experiments. Different ALE meta-analyses were carried out. One involved the subsets of studies involving meditation induced through exercising focused attention (FA). The network included clusters bilaterally in the medial gyrus, the left superior parietal lobe, the left insula and the right supramarginal gyrus (SMG). A second analysis addressed the studies involving meditation states induced by chanting or by repetition of words or phrases, known as “*mantra*.” This type of practice elicited a cluster of activity in the right SMG, the SMA bilaterally and the left postcentral gyrus. Furthermore, the last analyses addressed the effect of meditation experience (i.e., short- vs. long-term meditators). We found that frontal activation was present for short-term, as compared with long-term experience meditators, confirming that experts are better enabled to sustain attentional focus, rather recruiting the right SMG and concentrating on aspects involving disembodiment.

## Introduction

Meditation is a complex cognitive task aiming at self-regulating the body and mind and is often associated with neurophysiological and psychological modifications (Cahn and Polich, 2006). Practicing meditation is considered a way of training the mind (Barinaga, 2003; Knight, 2004). Meditation-related cognitive and physiological mechanisms involving refining the attention, enhancing attention skills, and developing very sophisticated means for investigating the nature of the mind from a first person perspective (Barinaga, 2003), have been consistently addressed by neuroscience regarding its potential benefit for mental and physical health (Davidson and McEwen, 2012). Accordingly, there has been a growing interest in brain imaging studies addressing the neural substrates of meditative brain states (Sperduti et al., 2011; Hasenkamp and Barsalou, 2012; Jerath et al., 2012; Tang et al., 2012; Vago and Silbersweig, 2012). There are many types of meditation practices eliciting different cognitive processes (e.g., silence, attention to own body, sense of joy, mantras, etc.). It is very possible that different practices of meditation are subserved by largely, if not entirely, disparate brain networks. Whether the cognitive state induced by the different meditation forms is the same is not known (Tang et al., 2012). Therefore, it is difficult to describe a meditation-related activation pattern independent of the practices needed to reach it.

One way to reach a meditation state is through chanting or repetition of sounds, words or phrases known as “*mantra*.” Mantra meditation is considered one of the most popular type of meditation and is present in many traditions (Braboszcz et al., 2010). A mantra is a sound, word, or sentence that can be either recited aloud or mentally, as internal speech. It is held that body vibrations and sensations induced by a mantra repetition help in calming and focusing the mind and the body without the need for intense concentrative efforts (Braboszcz et al., 2010) and are believed to result in profound relaxation, marked by breath quiescence, and reduced conceptual content (Travis et al., 2010). When meditators repeat the mantra, they are instructed to focus their attention on the recitation, or, at variance on its meaning if it has one. Some practices involve mantra repetition with awareness of the breath (and others without breath awareness) (Wang et al., 2011). Both Kundalini yoga and Acem meditators normally use mantras during meditation. This silent repetition of a short sequence of words such as sat nam is used as a key to achieve a meditative state of mind (Wang et al., 2011). fMRI studies addressing the neural correlates of mantra-induced meditation require participants to meditate (using a silent mantra) and, as control task, to silently repeat a short phrase, e.g., “table and chairs” (Engstrom et al., 2010). This neutral phrase is selected to not evoking an emotional response. In addition the control phrase is used in order to subtract language-related activations originating from the mantra repetition during meditation. It has been shown that mantra-meditation triggers activations in the inferior frontal gyrus bilaterally (Davanger et al., 2010), the medial prefrontal cortex, anterior cingulated cortex, limbic and superior parietal areas (Wang et al., 2011), or the hippocampus, middle cingulate cortex, and precentral cortex bilaterally are reported (Engstrom and Soderfeldt, 2010). It has been argued that during mantra repetition, there are some signs of meditation such as a mixed occurring of relaxed mantra repetition and spontaneously occurring thoughts, attempts to gently shifting back attention to the mantra when one becomes aware of mind wandering, with physical relaxation or stress reduction experiencing and an increased ability to accept and tolerate symptoms of stress as a normal part of meditation as well as everyday life (Davanger et al., 2010).

A further practice is concentration meditation, referred to as focused attention (FA), which is exercising the regulation of attention and executive frontal functions (Cahn and Polich, 2006; Lutz et al., 2008a). Meditation practices have been divided into two categories: FA meditation, which entails voluntary and sustained attention on a chosen object, on the breath or on different body parts, and open monitoring (OM) meditation, which involves non-reactive monitoring of the moment-to-moment content of experience (Lutz et al., 2004). A recent model of FA has been recently developed (Lutz et al., 2008b; Hasenkamp et al., 2012) and posits its main focus on exercising attentional control. OM of the content of the experience on the present moment (Conze, 2003) appears to be associated with brain regions involved in vigilance, monitoring, and disengagement of attention from sources of distraction (Lutz et al., 2008b). OM practices are based on an attentive set characterized by an open presence and a non-judgmental awareness of sensory, cognitive, and affective experience in the present moment (Cahn and Polich, 2006; Lutz et al., 2008b). Mindfulness of breathing elicits activations in the dorsal medial prefrontal cortex bilaterally and in the rostral anterior cingulate cortex (Holzel et al., 2007). During this practice, activations in bilateral dorsal anterior cingulate cortex and right medial anterior prefrontal cortex, and deactivations in the middle frontal gyrus, dorsolateral prefrontal cortex, precuneus, superior temporal gyrus, insula have been found (Manna et al., 2010).

Lastly, meditation based on exercising loving-kindness-compassion is held to create a general sense of well-being and to aid in prevention of feelings of anger or irritation (Lutz et al., 2007; Braboszcz et al., 2010). This practice is based on evoking feelings of compassion for a respected, a beloved and a neutral person internally visualized. Then the feelings are gradually extended toward a combination of persons and finally toward all living beings and everyday life. The final scope is developing a non-referential, reflexive state of compassion (Braboszcz et al., 2010). It has been shown that loving-kindness-compassion meditation increases activation in limbic regions, amygdala, right temporo-parietal junction, and right superior temporal sulcus (Lutz et al., 2008a). Benevolence and compassion trigger a significant positive coupling of heart rate (HR) and BOLD signal in the right middle insula, the dorsal anterior cingulate area, somatosensory cortices, and in the right inferior parietal lobule (Lutz et al., 2009).

Despite differences in cognitive processes and in brain activations, common components such as attention regulation (Naranjo and Ornestein, 1970; Cahn and Polich, 2006) and the detachment from one's own thoughts by means of a fusion between the subject and the object of meditation, and shared activations across different meditation practices have been reported (Newberg and Iversen, 2003; Rubia, 2009; Sperduti et al., 2011; Wang et al., 2011). In a meta-analysis including 10 meditation studies (Sperduti et al., 2011), common activations were reported in the basal ganglia, the enthorinal cortex, and medial prefrontal cortex. Surprisingly, no activation in the attentional networks, neither in areas related to body representation and interoception, often reported in the literature (Cahn and Polich, 2006; Tagini and Raffone, 2010), has been found. In addition, no deactivation pattern has been investigated, although meditation is known to change resting state activity (Farb et al., 2007; Pagnoni et al., 2008; Bærentsen et al., 2010; Manna et al., 2010; Taylor et al., 2011; Hasenkamp et al., 2012). In that meta-analysis (Sperduti et al., 2011), it has been suggested that attentional and cognitive control networks are not a key network subserving meditation and that previously reported frontal activations were possibly differently localized and consequently did not converge. Another possibility is that areas related to attentional mechanisms, as well as those related to body representation and interoception, are activated only by a subgroup of meditation practices. Given that, in the previous meta-analysis which encompassed 10 studies that included data from very diverse meditation practices, e.g., Yoga or Tantric, Acem, Kundalini Yoga, Mindfulness, Samatha, Vipassana Tibetan Buddhist, see Table 1 of Sperduti et al.'s paper (2011), frontal, parietal, as well as insular activations, related to attentional, body representation, and interoception mechanisms, were not reported. Therefore, to test whether areas related to attentional mechanisms, as well as those related to body representation and interoception are activated only by a type of meditation practice, in a first ALE meta-analysis we draw together imaging results from all relevant fMRI studies of meditation, with the goal of determining the range and extent of brain regions implicated. This first ALE analysis will evidence the core cortical network subserving meditation, since some common processes should be shared by all meditative techniques despite differences between meditation practices. However, the present analysis differs from the prior efforts in that it includes a larger number of studies (26, i.e., more than doubling the statistical power, by including data from 150 activation foci), as well as assesses negative signal changes (Raichle, 1998) which were completely neglected in the previous efforts. In addition, as a new feature with respect to previous efforts and to account for the above mentioned heterogeneity of experimental designs, we considered that the different meditation practices trigger different cognitive processes. We thus considered what the participants actually performed in the scanner behaviorally to reach the meditation state, by addressing two of the practices used to reach meditation states, i.e., the cognitive state induced by chanting or repetition of words or phrases known as “*mantra*,” and the state induced through FA. These two groups of studies were selected, being the only ones with sufficient information available in order to perform ALE meta-analysis. Interestingly, an fMRI study comparing the two practices evidenced that, limbic structures, insula, and lateral frontal areas were differentially activated by the FA practice, while the precentral gyrus, parietal cortex, and medial frontal gyrus were differentially activated by mantra repetition (Wang et al., 2011). Therefore, in a second ALE analysis we investigated whether these differences in activations related to attentional, body representation, and interoception mechanisms will be consistently dissociated across FA-based practice and mantra repetition induced meditation studies. Lastly, in interpreting the lack of frontal activation observed in the previous meta-analysis (Sperduti et al., 2011), authors argued that meditation expertise might have contributed to, since only studies recruiting expert practitioners were included. Based upon previous studies, reporting an inverted U-shape relation between frontal activity and meditators expertise (Brefczynski-Lewis et al., 2007), with experts showing less frontal activity, it might be predicted that, in the last meta-analysis, by further subdividing the included meditation studies and grouping them according to short-term and long-term meditation experience, we may determine the effect of expertise on frontal activations and on the meditation network.

**Table 1:** **Publications included in the meta-analysis, task they employed, number of subjects that were investigated and number of selected foci for the ALE meta-analysis; details of years of meditation experience**.

**N#**	**Study**	**Participants**	**Scanner**	**Contrast**	**Foci**	**Experience**	> **or** <**5000 h**
1	Bærentsen et al., 2010	31	3T	Meditation type 1 vs. rest	13 activations 12 deactivations	11 years	>
2	Bærentsen et al., 2010	21	3T	Meditation type 2 vs. rest	1 activations 33 deactivations	11 years	>
3	Brefczynski-Lewis et al., 2007	14	3T	Meditation vs. rest	15 activations	10.000–54.000 h	>
4	Davanger et al., 2010	4	1.5T	Meditation vs. control	2 activations	23 years	>
5	Engstrom et al., 2010	8	1.5T	Meditation vs. control	4 activations	14 months	<
6	Farb et al., 2007	16	3T	Meditation vs. control		8 weeks	<
7	Hasenkamp et al., 2012	15	3T	Meditation aware vs. rest	7 activations	>1 year	<
8	Hasenkamp et al., 2012	15	3T	Meditation shift vs. rest	6 activations	>1 year	<
9	Hasenkamp et al., 2012	15	3T	Meditation focus vs. rest	1 activations	>1 year	<
10	Holzel et al., 2007	15 meditators 15 non-meditators	1.5T	Meditation vs. control in experts vs. ctr.	6 activations	7.9 years	<
11	Ives-Deliperi et al., 2011	10	3T	Meditation vs. control	1 activations 7 deactivations	4 years	<
12	Khalsa et al., 2009	11	SPECT	Meditation vs. rest	6 activations 15 deactivations	–	<
13	Kalyani et al., 2011	4/12 meditatiors 8/12 naive	3T	Meditation vs. control	13 deactivations	–	<
14	Lazar et al., 2000	5	3T	Meditation vs. control	15 activations	4 years	<
15	Lou et al., 1999	9	PET	Meditation type 1 vs. rest	11 activations	>5 years	<
16	Lou et al., 1999	9	PET	Meditation type 2 vs. rest	3 activations	>5 years	<
17	Lutz et al., 2008a	16	3T	Meditation vs. rest	10 activations	10.000–50.000 h	>
18	Lutz et al., 2009	10 meditators 13 non meditators	3T	Meditation vs. rest in expert vs. novices	2 activations	10.000–50.000 h	>
19	Manna et al., 2010	8		Meditation type 1-rest	3 activations 11 deactivations	15,750 h	>
20	Manna et al., 2010	8		Meditation type 2-rest	3 activation	15,750 h	>
21	Shimomura et al., 2008	8	1.5T	Meditation type 1-rest	6 activations	10 years	>
22	Shimomura et al., 2008	8	1.5T	Meditation type 2-rest	6 activations	10 years	>
23	Pagnoni et al., 2008	12	3T	Meditation vs. rest	8 activations	>3 years	<
24	Taylor et al., 2011	12	3T	Meditation vs. rest in experts vs. novices	2 deactivations	>1000 h	<
25	Wang et al., 2011	10	3T	Meditation type 1-control	2 activations 2 deactivations	30 years	>
26	Wang et al., 2011	10	3T	Meditation type 2-control	5 activations 4 deactivations	30 years	>

## Methods

### Data used for the meta-analysis

Functional imaging studies included in the meta-analysis were obtained from an exhaustive PubMed-, ISI web of knowledge-, and the Cochrane literature-search (strings: “meditation,” “fMRI”) on neuroimaging experiments. The literature cited in the obtained papers was also assessed to identify additional neuroimaging studies pertaining to meditation.

We included studies based on the following inclusion criteria. Subjects were neurologically healthy adults and experiments required participants to perform meditation as task during MRI measurements. As there is no clear universal definition of meditation, in selecting the studies we closely followed the meaning of meditation used by the authors of the included studies. Studies not employing meditation fMRI tasks in the scanner were also not included because they did not measure functional activity during meditation, but rather during other fMRI tasks. The field of view covered the whole brain and results were reported in a standard reference space (Talairach/Tournoux, MNI). Differences in coordinate spaces (MNI vs. Talairach space) were accounted for by transforming coordinates reported in Talairach space into MNI coordinates using a linear transformation (Lancaster et al., 2007). Analyses were not restricted to regions of interest, studies were only considered if they reported results of whole-brain group analyses; a random-effects analysis was performed, and single-subject reports were excluded. We excluded studies not reporting results of whole brain group analyses as coordinates in a standard reference space. Anatomical studies showing structural changes (*N* = 8) have been excluded (Lazar et al., 2005; Pagnoni and Cekic, 2007; Holzel et al., 2008, 2010, 2011; Luders et al., 2009; Vestergaard-Poulsen et al., 2009; Grant et al., 2010), as well as studies addressing connectivity (*N* = 5) (Guo and Pagnoni, 2008; Brewer et al., 2011; Jang et al., 2011; Josipovic et al., 2011; Kilpatrick et al., 2011). Selected fMRI contrasts have been kept as homogenous as possible (i.e., meditation vs. rest). However, when this was not possible, we included those which were presented in the selected papers [meditation vs. control task, e.g., arithmetic (Holzel et al., 2007)]. Obviously, inserting different types of contrasts, other that task > rest, is a common aspect in published ALE-meta-analysis, as there is variability in the type of control tasks used in the different fMRI, e.g. (Caspers et al., 2010). For instance, in a previous meta-analysis on meditation, aside including meditation > rest contrasts, also meditation > control contrasts, which included pseudowords and words repetition, silent repetition of words or numbers, or random generation of numbers, or animals, have been used (Sperduti et al., 2011). The inclusion of experts meditators was applied in all except for one study (Farb et al., 2007) in which data from mindfulness meditators who trained in an 8-week intensive course (Kabat-Zinn, 2003) were not excluded from the analysis due to the importance of including data from a comparison on two meditation practices, e.g., experiential focus vs. narrative focus meditation; three studies (Holzel et al., 2007), (Lutz et al., 2009), (Taylor et al., 2011) only reporting between groups comparisons were also included since the reported activation clusters are driven by meditation in expert meditators only.

The ALE analyses were conducted on 24 fMRI meditation studies which included data from 275 subjects. Based on the above mentioned criteria, 19 articles (reporting 16 fMRI, 1 PET, and 1 SPECT studies) were designated as suitable for the first general meta-analysis (see Table 1). The total number of experiments included was 26, since six studies reported coordinates for more than one contrast. In this case, all of the contrasts were included in the meta-analysis as a separate dataset from the same study since all reflected meditation related activations (Table 1). Together, the selected studies included data from 329 subjects and reported 24 experiments with 150 activation foci. Five ALE analyses were carried out: the “Meditation Network: Activations” analysis included all of the eligible studies, in order to assess the general meditation brain network, by determining brain areas with consistent activation across all studies on meditation considered together. Similarly in the “Meditation Network: Deactivations” we determined brain areas with consistent deactivation across all studies on meditation considered together. Deactivations during meditation, which is synonymous with “activation during rest” (Raichle, 1998; Bærentsen et al., 2010) were identified in a separate analysis including those studies that reported results of whole brain group analyses as coordinates for the contrast rest vs. meditation (163 subjects and reported 11 experiments with 103 activation foci, see Table 1 where deactivations have been reported). The ALE meta-analyses show that significant results are achieved if convergence across meditation studies occurs, more likely than expected, by chance, even though this does not require all or even the majority of the meditation studies to activate a particular area (Eickhoff et al., 2009, 2011). Considering the different cognitive processes underlying the different meditation practices, these components may influence the analysis across the whole sample of meditation experiments. To explore the effects of these potential arguments, the reported studies were grouped as follows in a further analysis: (1) the cognitive state induced by chanting or repetition of words or phrases known as “*mantra*” (9 experiments, 106 subjects, and 62 activation foci) and (2) meditation induced through FA (13 experiments, 197 subjects, and 91 activation foci). In addition, in a last analysis (“long- and short-term meditation experience”), we divided studies according to experience (see Table 1), with long-term experience >5000 h (7 experiments, 102 subjects, and 48 activation foci) and short-term experience arbitrarily set roughly at <5000 h/10 years meditation practice (9 experiments, 161 subjects, and 48 activation foci). The analysis performed on the short-term meditation experience group was performed a second time by excluding data from studies involving participants with less than 1 year meditation experience.

### Statistical procedure

A statistical map was generated by using a collection of foci after transferring these foci into MNI space (Lancaster et al., 2007). Meta-analysis was completed using the revised version (Eickhoff et al., 2009, 2011) of the activation likelihood estimation (ALE) approach for coordinate-based meta-analysis of neuroimaging results (Turkeltaub et al., 2002; Laird et al., 2005, 2009). To account for the uncertainty that is technically inherent to the actual location of the peaks, each coordinate was modeled not as a single point, but by a three-dimensional (3D) Gaussian function with 12 mm FWHM (Laird et al., 2005, 2009; Eickhoff et al., 2009). Thus, the localization probability distributions describe the probability that a given focus actually lay within a particular voxel (Laird et al., 2005, 2009; Eickhoff et al., 2009, 2011). Statistical significance is gained utilizing a permutation test of randomly generated foci, using the same FWHM and number of foci. The voxel-wise comparison is tested against the null-hypothesis of uniformly distributed peaks, giving a set of ALE-values necessary for thresholding the probability map. Using the False Discovery Rate (FDR) with *q* = 0.01, the test was corrected for multiple comparisons (Laird et al., 2005, 2009; Eickhoff et al., 2009, 2011) and a minimum cluster size of 100 mm^3^ was set. The resulting areas were anatomically labeled by reference to probabilistic cytoarchitectonic maps of the human brain using the SPM Anatomy Toolbox (Eickhoff et al., 2005). Using a Maximum Probability Map (MPM), activations were assigned to the most probable histological area at their respective locations.

## Results

### Meta-analysis across all included studies

#### Activations

**Figure 1:**
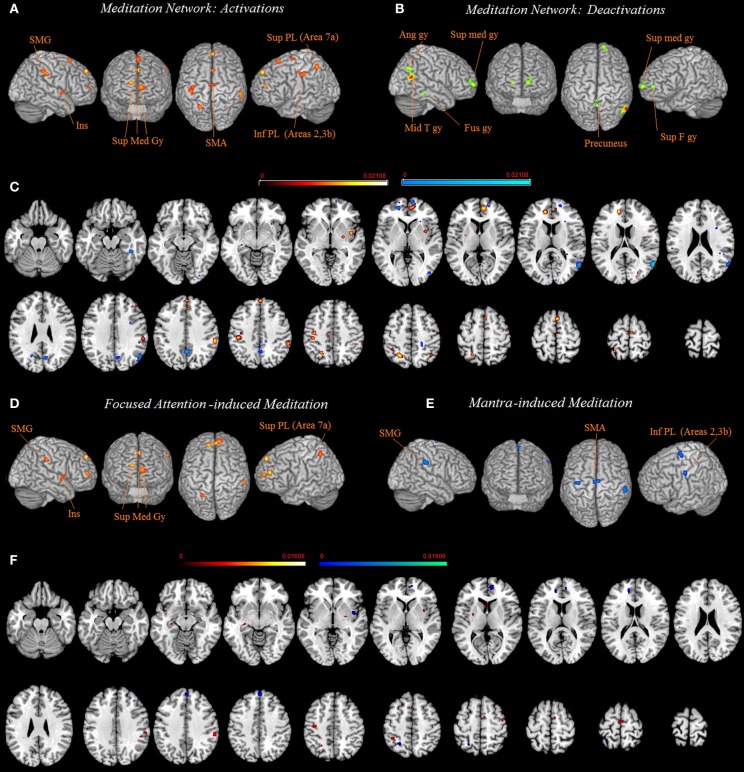
**Common network of activations (A) and deactivations (B) underlying meditation are displayed on a rendered template brain provided by spm5 and on axial slices of the MNI single subject template, with activations (in orange) and deactivations (in blue) overlaid on the same template (C).** Relative increases in neural activity associated with meditation induced trough focused attention **(D)** and through mantra recitation **(E)** are displayed on a rendered template brain provided by spm5 and on axial slices of the MNI single subject template, with FA related activations (orange) and mantra recitation related activations (blue) overlaid on the same template **(F)**. All activations are significant at *p* < 0.05 corrected for multiple comparisons using the false discovery rate (FDR). Color bas show *ALE* value.

The 10 activation clusters resulting from the meta-analysis of all the included studies comprised bilaterally the superior medial gyrus (clusters 1 and 6), more superiorly the left superior medial gyrus (cluster 5), medially the left paracentral lobule (cluster 10) and the right supplementary motor area (SMA) (hereafter SMA, clusters 7 and 9), the left superior (Area 7a, cluster 3) and inferior parietal lobe (Area 2, extending to area 4p and 3b, cluster 4), and left insula (cluster 8). Right lateralized activation was found in the supramarginal gyrus (SMG) (cluster 2) (Table 2 and Figure 1).

**Table 2:** **Results from the ALE meta-analysis**.

**Cluster**	**Area**		**MNI coordinates**			**Cluster size (voxels)**	**ALE Max**
			***x***	***y***	***z***		
**MEDITATION NETWORK: ACTIVATIONS**							
1	R	Superior medial gyrus	8	50	6	95	0.013
2	R	Supramarginal gyrus	58	−34	36	86	0.012
3	L	Superior parietal lobe (Area 7a)	−24	−58	50	83	0.012
4	L	Inferior parietal lobe (Area 2)	−42	−28	42	81	0.011
	L	Postcentral gyrus (Area 4p, 3b)	−38	−18	44		0.010
5	L	Superior medial gyrus	2	48	38	56	0.016
6	L	Superior medial gyrus	−12	44	14	51	0.015
7	R	Supplementary motor area	2	14	60	48	0.011
8	R	Insula	42	0	−4	38	0.010
9	R	Supplementary motor area	4	−14	64	28	0.010
10	L	Paracentral lobule	−4	−16	64	95	0.009
**MEDITATION NETWORK: DEACTIVATIONS**							
1	R	Middle temporal gyrus	58	−62	16	112	0.021
2	R	Superior medial gyrus	12	62	0	65	0.012
	R	Superior frontal gyrus	18	56	8		0.010
3	R	Precuneus	2	−56	38	65	0.015
	L	Precuneus	−2	−46	38		0.009
4	R	Angular gyrus	52	−66	32	45	0.012
5	R	Precuneus	8	−68	28	36	0.012
6	R	Fusiform gyrus	40	−40	−20	29	0.012
7	L	Superior medial gyrus	−18	52	0	29	0.012
**FOCUSED ATTENTION INDUCED MEDITATION**							
1	R	Superior medial gyrus	8	50	6	95	0.013
2	L	Superior parietal lobe (Area 7a)	−24	−58	52	57	0.011
3	L	Superior medial gyrus	2	48	38	56	0.016
4	L	Superior medial gyrus	−12	44	14	54	0.015
4	R	Insula	42	0	−4	38	0.010
5	R	Supramarginal gyrus	58	−32	34	43	0.011
**“MANTRA” INDUCED MEDITATION**							
1	R	Supramarginal gyrus	58	−32	34	62	0.011
2	R	Supplementary motor area	4	−14	64	54	0.009
	L	Paracentral lobule	4	−16	64		0.009
3	L	Postcentral gyrus (Area 4p, 3b)	−36	−18	44	37	0.010
**LONG EXPERIENCE**							
1	R	Supramarginal gyrus	58	−34	36	99	0.012
2	R	Supplementary motor area	2	14	60	67	0.011
3	L	Superior parietal lobule (Area 7a)	−22	−60	50	64	0.010
4	R	Supplementary motor area	4	−14	64	43	0.009
	L	Paracentral lobule	−4	−16	64		0.009
5	L	Post/pre central gyrus (Area 4p)	−36	−18	44	34	0.010
6	R	Middle cingulate	6	30	36	25	0.009
**SHORT EXPERIENCE**							
1	R	Superior medial gyrus	8	50	6	67	0.012
2	L	Superior medial gyrus	−12	44	14	66	0.015
3	L	Superior medial gyrus	2	48	40	66	0.016
4	L	Inferior parietal lobule (Areas 2, 3b)	−42	−28	42	57	0.11
5	L	Superior parietal lobule (Area 7)	−30	−50	66	25	0.009

Peaks of activation corrected above the threshold, MNI coordinates (x, y, and z) of maximum ALE-value, and maximum ALE-value of this cluster. All peaks are assigned to the most probable brain areas as revealed by the SPM Anatomy Toolbox (Eickhoff et al., 2005).

#### Deactivations

The seven clusters resulting from the meta-analysis of deactivation results (analysis 2) included bilaterally the precuneus (clusters 3 and 5) and the superior medial gyrus (clusters 2 and 7). In addition, a right lateralized network of deactivated areas included the middle temporal gyrus (cluster 1), the angular gyrus (cluster 4), and the fusiform gyrus (cluster 6) (Table 2 and Figure 1).

### Subdivision

#### “Mantra”-induced meditation and focused attention-induced meditation

Practices involving chanting or repetition of words or phrases, known as “mantra,” included clusters of activity in the right SMG (cluster 1), the right SMA and paracentral lobule (cluster 2), and the left postcentral gyrus (Areas 4p, 3b, cluster 3). The network supporting the forms of meditation induced through FA, included clusters bilaterally in the medial gyrus (clusters 1, 3, and 4), the left superior parietal lobe (cluster 2), the left insula (cluster 4) and the right SMG (cluster 4) (Figure 1 and Table 2).

#### Short- and long-term meditation experience

**Figure 2:**
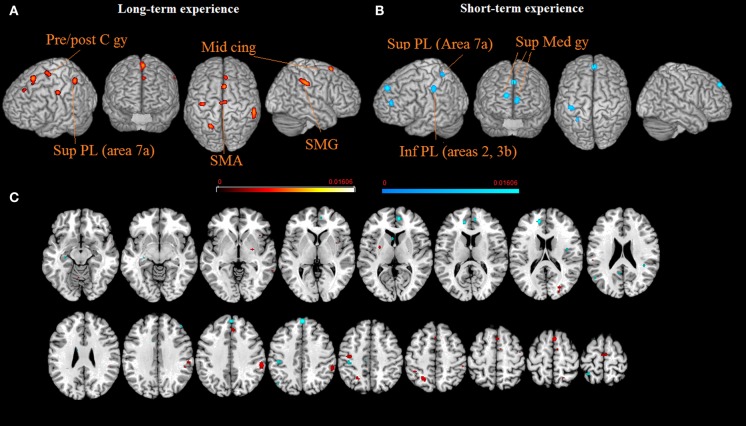
**The brain networks supporting long-term (A) and short-term (B) meditation experience are displayed on a rendered template brain provided by spm5 and on axial slices of the MNI single subject template, with long-term (orange) and short-term (blue) meditation experience related activations overlaid on the same template (C).** All activations are significant at *p* < 0.05 corrected for multiple comparisons using the false discovery rate (FDR). Color bas show *ALE* value.

Lastly, in further sub-analysis we investigated the effects of meditation experience. The brain network supporting long experience meditation included cluster of activity in the right SMG (cluster 1), the SMA and the paracentral lobule bilaterally (clusters 2 and 4), the middle cingulated cortex and the postcentral gyrus (clusters 5 and 6). Short experience included clusters of activity in the superior medial gyrus and the inferior parietal lobule (clusters 1, 2, and 3, Table 2 and Figure 2). By contrast, the superior parietal lobe was found activated independent of meditation experience. By excluding from the analysis performed on the short-term meditation experience data from studies involving participants with less than 1 year meditation experience, we found the same pattern of results.

## Discussion

We performed a series of quantitative meta-analyses (Turkeltaub et al., 2002; Laird et al., 2005, 2009; Eickhoff et al., 2009) of the current literature on meditation, by including a total of 26 imaging experiments, with 329 subjects and 150 activation foci.

Activation related to meditation was found with a network involving frontal and parietal areas bilaterally, and the right insular cortex. We found that some coordinates for several regions appeared twice, e.g., for the “Meditation Network: Activations” analysis including all of the eligible studies and for the further subdivisions, e.g., the “long- and short-term meditation experience” analysis. The ALE meta-analyses show that significant results are achieved if convergence across meditation studies occurs, more likely than expected, by chance, even though this does not require all or even the majority of the meditation studies to activate a particular area (Eickhoff et al., 2009, 2011). Therefore, some activation clusters may be driven mainly by a subgroup of studies included in the meta-analyses. This pattern of results is commonly found in previous ALE meta-analysis, e.g., Caspers et al. (2010), where authors subdivided the studies included in the main analysis into several subgroups, which logically involved a different and smaller sample (i.e., number of subjects and number of activation foci), and found that some clusters appeared twice, and in some cases three times, in the general analysis first, and then in the sub-analyses. These results are all similar to our results. By using the ALE method the comparison of activations between groups of studies is done on thresholded maps. Accessing the raw results, by performing image-based meta-analyses would strengthen the results, since such approaches use more information from the original data despite their low applicability (Caspers et al., 2010). However, a recent comparison of image- and coordinate-based meta-analyses (Salimi-Khorshidi et al., 2009) revealed good agreement between metaanalyses based on full statistical contrast images and reduced 3D coordinates. Nonetheless, meta-analysis results would definitely benefit from data sharing initiatives, e.g., Schilbach et al. (2008).

### Meditation network: activations

As far as the parietal cortex is concerned, meditation-related activity was found in the left superior parietal lobe (Area 7a, cluster 3). This activation can be related to egocentric (body- and body part-centered) coordinates coding (Makin et al., 2007), to the processing of multimodal integrated spatial representations in body-centered coordinates (Felician et al., 2004) and to updating postural representations of the upper limb (Pellijeff et al., 2006). In a previous study, some of us found that the left posterior IPS codes an allocentric, but not egocentric, visual model of the body (the body structural description) (Corradi-Dell'Acqua et al., 2009). Taken together, these studies suggest that a left area 7a activation may reflect a continuous alteration or updating of egocentric and allocentric coordinates. Area 2 of the left inferior parietal lobe was also part of the meditation network (cluster 4). It is a somatosensory area and its activation is consistent with practicing the body-scan that is experienced in some forms of meditation practices. This area is involved in the computation of the relative size and shape of body parts, in perceiving changes in the relative size of body parts induced by a perceptual illusion (Ehrsson et al., 2005), and during imagery of contralateral body part movements (Wolbers et al., 2003). The parietal cluster also included area 3b (cluster 4) which is responsible for touch representation. This activation could also be related to the technique used in several meditation practices where participants touched fingers two to five to the thumb, according to a particular rhythm (Khalsa et al., 2009).

Activation in the right SMG (cluster 2) is similar to activation found during disembodiment (De Ridder et al., 2007). An altered integration of visual, tactile, proprioceptive, and vestibular information, which takes place in the right temporo-parietal junction, may trigger disembodiment (Blanke and Mohr, 2005). Furthermore, this area is part of an attention network (Corbetta and Shulman, 2002).

The meditation network included the right insular cortex (cluster 8), which supports a representation of somatic and visceral responses accessible to consciousness (Critchley et al., 2004), consistent with practicing conscious contemplation in the body, i.e., body scan. This area is richly interconnected with regions involved in autonomic regulation (Cechetto, 1994), and has a topographical representation of inputs from visceral, olfactory, gustatory, visual, auditory and somatosensory areas to integrate representations of external sensory experience and internal somatic state (Augustine, 1996).

In the frontal cortex, we found aactivations in the superior medial gyrus and in a more superior part of the left superior medial gyrus (clusters 1, 5, and 6). This area has been related to emotional processing (Phan et al., 2002), self-referential processing (Northoff et al., 2006), and is part of a distributed attention network (Corbetta and Shulman, 2002). It is suggested that during meditation subjects are improving their emotion regulation (Holzel et al., 2007). Emotional self-regulation strategies, such as cognitive distancing and reappraisal (Beauregard et al., 2001; Levesque et al., 2003), deciding whether an adjective is referred to the self (van der Meer et al., 2010), evaluating self-referential stimuli (Northoff and Bermpohl, 2004), memory for self-traits (Kelley et al., 2002), reflected self-knowledge (Lieberman et al., 2004), aspirations for the future (Johnson et al., 2006), or first person perspective taking (David et al., 2006) have all been found to activate the medial prefrontal cortex. Thus, the mPFC is involved in the implicit representation of the self and in linking subjective experiences across time (Northoff et al., 2006).

Lastly, activity in the right SMA (clusters 7, 9 and 10) was found in the general analysis. Meditation on the body parts demonstrated strong activation of the SMA (Lou et al., 1999). This area is activated by passive arm movements (Weiller et al., 1996). In addition, SMA activation has been found during internal simulation of kinesthetic sensation during motor imagery (Naito et al., 2002) and during temporal processing, i.e., the mechanisms that underlie the encoding, decoding, and evaluation of temporal structure (Schwartze et al., 2012). The ability to assess temporal structure is crucial in order to adapt to an ever-changing environment.

### Negative signal changes involved in meditation

The subtraction of the meditation condition from a control task reveals brain areas in which neural activity decreases during meditation performance, reflecting the “default network” (DMN) (Raichle, 1998), which has been described as a set of regions which are “activated” during rest and “deactivated” during cognitively effortful tasks. We found meditation related negative signal changes in the right hemisphere in the fusiform, angular, and middle temporal gyri, and in medial structures of the precuneus and the superior medial gyrus. The occipital lobe is activated during mental imagery (Kosslyn et al., 2001), and it has been suggested that its deactivation is associated with the subjective experience of the mind becoming quieted (Khalsa et al., 2009). Deactivation of the angular gyrus has been interpreted as the product of an increase of the self-trascendence (Newberg and Iversen, 2003; Urgesi et al., 2010) and as an enhancement of visuospatial attention (Archer et al., 2003). Together with the middle temporal regions, the angular gyrus and the precuneus are part of the DMN (Raichle, 1998). DMN activation decrease determines a reduction during meditation of the mind wander in the mental mind traveling (Schacter et al., 2007) bringing the subject back to the present moment experience. The precuneus has been associated to self-referential processing (Cavanna and Trimble, 2006) and its deactivation is consistent with one of the aims of meditation practice that is the reduction of the self (Manna et al., 2010). Lastly, we found that parts of the medial frontal gyrus were activated and parts of it deactivated confirming that meditative states may be associated to deactivation (Dietrich, 2003; Lou et al., 2005), or activation (Cahn and Polich, 2006) in executive networks (Manna et al., 2010). It has been shown that FA enhances (predominantly right) medial frontal and reduces (predominantly left) lateral prefrontal area, whereas OM meditation activates the (predominantly left) medial frontal area (Manna et al., 2010).

### Focused attention induced meditation network

FA induced meditation triggered activation in the superior medial gyrus and in a more superior part of the left superior medial gyrus (clusters 1, 3, and 4), an area known to be involved in the implicit representation of the self and in linking subjective experiences across time (Northoff et al., 2006), as discussed above. Interestingly our coordinates of the medial cluster were very close to those activated in a conjunction analysis both for the DMN and for the self processing (*x* = 2, *y* = 52, *z* = 0) (Whitfield-Gabrieli et al., 2011) In addition, attentional focus is therefore an important element of meditation (Newberg and Iversen, 2003; Cahn and Polich, 2006). Executive attention (Posner and Petersen, 1990), sustained attention (Frith et al., 1991; Pardo et al., 1991), and cognitive control (Carter et al., 1995; MacDonald III et al., 2000) have been shown to activate the prefrontal area. In line with these results, our meta-analysis confirmed that activations of the medial frontal cortex are related to meditation which is known to induce an inward shift of attention and an increased awareness of inner states. Accordingly, this area was found activated specifically during FA induced meditation.

FA induced meditation triggered activation in the right SMG (cluster 5). This activation might be related to disembodiment (De Ridder et al., 2007) and to attention (Corbetta and Shulman, 2002), which are both functions exercised during FA meditation.

In the sub-analysis on FA-induced meditation, the superior parietal area 7a was found activated (cluster 2). Aside being related to egocentric (body- and body part-centered) coordinates coding (Makin et al., 2007) as discussed in the previous paragraph, it has been suggested that this region may be altered with extended daily FA to moment-to-moment experience and thus may represent the neural underpinnings of self reference in the psychological present (Farb et al., 2007).

Lastly, the insular cortex (cluster 4) was also found activated during FA-based meditation. Particularly relevant for the present study is the role of the insular cortex in corporeal awareness (Berlucchi and Aglioti, 1997) and heightened self-awareness (Landtblom et al., 2011). For example, it has been shown that stimulation of the insular cortex triggers illusions of changes in body position and out-of-body-experiences (Penfield and Faulk, 1955), that insular lesions provoke somatic hallucinations (Roper et al., 1993), and that the posterior insula is a critical lesion site in patients suffering from anosognosia for hemiparesis (Cereda et al., 2002). Lastly, the phenomenal incorporation of a rubber hand into the body schema (Tsakiris et al., 2012) results in activation of the insular cortex. Activation in the insular cortex was found specifically in the sub-analysis on FA-induced meditation. It has also been suggested (Farb et al., 2007) that awareness of momentary self-reference is derived from neural markers of transient body states, in particular, right lateralized exteroceptive somatic and interoceptive insular cortices (Critchley et al., 2004). It has been suggested that among other regions, the insular cortex likely represents the contents of the present-focused awareness, being associated with feedback regarding the interoceptive physiologic, exteroceptive somatic condition of the body, and overall corporeal awareness (Farb et al., 2007).

### Mantra-induced meditation network

Activation in area 3b was found specifically in the sub-analysis on mantra-induced meditation (cluster 3). This parietal area is responsible for touch representation. This activation could be related to the technique used in several meditation practices where participants touched fingers two to five to the thumb, according to a particular rhythm (Khalsa et al., 2009), especially in mantra-based meditation practices.

Mantra induced meditation triggered activation in the right SMG (cluster 1). Activation in this area was found both in the sub-analysis on FA induced meditation and in that on mantra induced meditation, meaning that activation in this area is shared by both practices. This activation might be related to disembodiment (De Ridder et al., 2007) and attention (Corbetta and Shulman, 2002).

Lastly, activity in the right SMA (cluster 2) was found in the mantra-induced meditation analysis. Aside the role of this area in internal simulation of kinesthetic sensation during motor imagery (Naito et al., 2002) and in the encoding, decoding, and evaluation of temporal structure (Schwartze et al., 2012), as discussed above, this activation could also be related to the technique used in several meditation practices where participants touched fingers two to five to the thumb, according to a particular rhythm (Khalsa et al., 2009).

### Short- and long-term meditation experience

As stated by meditators, regular meditation practice enables them to focus their attention on a single object for an extended period of time, and that distractions disturb this focus less frequently. It is possible that the increased ability to disregard distractions might cause a diminished need for attention-related activation (Holzel et al., 2007). Middle cingulate activation was present for short-term experience meditation group. The present analysis did not replicate the finding of activations in the anterior cingulated cortex (Holzel et al., 2007), confirming that activation in this area may be dependent upon differences in type of meditation, and on the amount of intentional effort required in target and baseline tasks, as it has previously been suggested (Bærentsen et al., 2010). Experienced meditators are typically less distracted and better enabled to sustain attentional focus (Brefczynski-Lewis et al., 2007). Accordingly, we found that medial frontal activation was present for short experience meditation groups (Farb et al., 2007). By contrast, we found that SMA and SMG activations were present for long-term experience meditation groups. These results might reflect expert meditators as using less strategies which are based on executive attention. Rather, they seem to concentrate on aspects mainly involving disembodiment (De Ridder et al., 2007). As far as the parietal cortex is concerned, independent of meditation experience, superior parietal lobule (Area 7a) activation was present for both long- and short-term experience meditation groups, indicating that the continuous updating of egocentric and allocentric coordinates is a common processing component. In addition, superior parietal lobule (Areas 2, 3b) activation suggested that the perception of changes in the relative size of body parts (Ehrsson et al., 2005) and motor imagery (Wolbers et al., 2003) elicited during body scan might trigger stronger activations in the short-term experience meditation group.

### Relating the ALE results with studies addressing structural and functional connectivity changes

Although the investigation about the relation between white-matter tract integrity, brain volume, and functional connectivity is just at the beginning, several recent meditation studies using these techniques found consistent results. Greater functional connectivity within the default mode network was found in the medial prefrontal cortex area for meditators practicing “Brain-wave vibration meditation” mind-body training as compared to controls (Jang et al., 2011). This practice enhances focusing attention on bodily sensations and emotions, and heightening awareness of the movement of energy within the body. Authors argued that results suggest that meditation practice may be associated with functional changes in regions related to internalized attention (Jang et al., 2011). Others (Brewer et al., 2011) found that the main nodes of the default-mode network (medial prefrontal and posterior cingulate cortices) were relatively deactivated in experienced meditators practicing several different meditations (Concentration, Loving-Kindness, Choiceless Awareness) as compared to meditation-naïve controls. This result is likely reflecting the effect of training attention away from self-reference and mind-wandering, and potentially away from the default-mode processing, which is common to all three of these meditation techniques. In addition, in the same study, a stronger coupling between the posterior cingulate, dorsal anterior cingulate, and dorsolateral prefrontal cortices (regions previously implicated in self monitoring and cognitive control), was found in experienced meditators (Brewer et al., 2011). Still, others (Hasenkamp and Barsalou, 2012) found that focused-attention experienced meditation increased functional connectivity with attentional networks, as well as between attentional regions and medial frontal regions. Authors argued that this pattern could be related to the increased ability to maintain attention and disengage from distraction that are reported for experienced mediators (Hasenkamp and Barsalou, 2012). It has also been shown that Mindfulness-Based Stress Reduction (MBSR) training alters intrinsic connectivity networks (Kilpatrick et al., 2011). Authors found increased connectivity between the mPFC and primary interoceptive awareness regions, including the posterior insula. Similarly (Farb et al., 2012) by using PPI analysis found that mindfulness training negative functional connectivity between DMPFC and the right posterior insula, authors suggested that this negative connectivity could serve to sustain positive activation in interoceptive representations area. These studies suggest that meditation-related changes is mediated by the modulation of the functional connectivity between brain regions. It is likely that that these activation changes persist. This possibility is supported by changes in structural connectivity, i.e., the white matter and in gray matter. It has been shown that fractional anisotropy (FA)—an indicator of white matter integrity—of several fiber tracts, including the superior and anterior corona radiata, the genu and body of the corpus callosum, and the superior longitudinal fasciculus can be increased by an Integrative Body–Mind Training (IBMT) training (Tang et al., 2010). Other authors found that the largest group structural connectivity differences in practitioners of Shamatha, Vipassana, and Zazen meditation styles, as compared to controls, were observed within the corticospinal tract, the temporal component of the superior longitudinal fasciculus, and the uncinate fasciculus (Luders et al., 2011). In particular, the uncinate fasciculus has a ventral part that connects the orbital cortex with the amygdala and the hippocampal gyrus. Thus, the larger fractional anisotropy of this tract may relate to the gray matter changes observed in the anterior part of the frontal lobe. It has been shown that meditators with different meditation traditions had larger gray matter volumes in the right orbito-frontal cortex and in the right hippocampus (as well as in the right thalamus and left inferior temporal gyrus when co-varying for age and/or lowering applied statistical thresholds) associated with emotional regulation, response control, and mindful behavior (Luders et al., 2009). Other studies examined differences in GM concentration (Holzel et al., 2008) and cortical thickness (Lazar et al., 2005) associated with insight meditation (Vipassana) which involves FA to internal experiences. Significantly increased GM concentration in the left inferior temporal gyrus, right anterior insula, right hippocampus, and right middle/superior frontal cortex (Holzel et al., 2008) and thicker prefrontal cortex and right anterior insula (Lazar et al., 2005) was found in meditators as compared to controls. These areas are associated with attention, interoception, and sensory processing. Other auhtors (Vestergaard-Poulsen et al., 2009) compared groups of highly experienced meditators practicing breathing based meditation and normal controls. In addition to find between-group differences in the lower brainstem, authors found increased gray matter densities in the left superior frontal gyrus and in the left inferior frontal gyrus, in the cerebellum bilaterally and in the left fusiform gyrus. Interestingly, a positive association between the gray matter volume in the right anterior insula and the right amygdala and the facet of mindfulness on the Five Facet Mindfulness Questionnaire (FFMQ) and (Murakami et al., 2012), which assess individual differences in mindfulness states and is composed of five facets: non-reactivity to inner experience, non-judging, acting with awareness, describing, and observing. This finding further supports the crucial role of the insula for the experience of a mindful state. Other authors (Leung et al., 2012) examined brain changes related to the practice of an emotion-oriented meditation: loving-kindness meditation (LKM) and found that meditators as compared with novices, had more gray matter volume in the right angular and posterior parahippocampal gyri. The right angular gyrus is important for affective regulation associated with empathic response, anxiety, and mood. Interestingly, in our meta-analysis this area was found deactivated. Deactivation of the angular gyrus has been interpreted as the product of an increase of the self-trascendence (Newberg and Iversen, 2003; Urgesi et al., 2010). By contrast, our meta-analysis showed that a region close to the angular gyrus, the right SMG, was found activated. Activation in this area was found both in the sub-analysis on FA-induced meditation and in that on mantra induced meditation, meaning that activation in this area is shared by both practices.

To sum up the above mentioned studies together with our meta-analysis support the view that focused-based meditation triggers both functional (as shown in the present meta-analysis) and structural changes (in the morphometric studies) particularly in two key areas, corresponding to the anterior part of the righ insula (Lazar et al., 2005; Holzel et al., 2008; Vestergaard-Poulsen et al., 2009) and the frontal lobe (Lazar et al., 2005; Holzel et al., 2008; Luders et al., 2009; Vestergaard-Poulsen et al., 2009). With respect to the latter, we found that parts of the medial frontal gyrus were activated and parts of it deactivated confirming that meditative states may be associated to deactivation as shown by the above mentioned connectivity studies, or activation (Cahn and Polich, 2006) in executive networks (Manna et al., 2010).

## Conclusions

The pattern of our meta-analysis highlights how meditation consistently triggers changes in executive attentive networks, in body representations and in areas processing interoceptive awareness information, as well as negative signal changes in the default brain network. Furthermore, the present study evidenced how activation in some areas are specifically driven by a subgroup of studies, dependent on the expertise and on the practices needed to obtain it.

### Conflict of interest statement

The authors declare that the research was conducted in the absence of any commercial or financial relationships that could be construed as a potential conflict of interest.
